# Marker-Based Estimates Reveal Significant Nonadditive Effects in Clonally Propagated Cassava (*Manihot esculenta*): Implications for the Prediction of Total Genetic Value and the Selection of Varieties

**DOI:** 10.1534/g3.116.033332

**Published:** 2016-08-30

**Authors:** Marnin D. Wolfe, Peter Kulakow, Ismail Y. Rabbi, Jean-Luc Jannink

**Affiliations:** * Department of Plant Breeding and Genetics, Cornell University, Ithaca, New York 14853; † International Institute of Tropical Agriculture (IITA), Ibadan, Oyo, Nigeria; ‡ United States Department of Agriculture - Agricultural Research Service (USDA-ARS), R.W. Holley Center for Agriculture and Health, Ithaca, New York 14853

**Keywords:** Genomic selection, Nonadditive effects, Cassava, GenPred, Shared Data Resources

## Abstract

In clonally propagated crops, nonadditive genetic effects can be effectively exploited by the identification of superior genetic individuals as varieties. Cassava (*Manihot esculenta* Crantz) is a clonally propagated staple food crop that feeds hundreds of millions. We quantified the amount and nature of nonadditive genetic variation for three key traits in a breeding population of cassava from sub-Saharan Africa using additive and nonadditive genome-wide marker-based relationship matrices. We then assessed the accuracy of genomic prediction for total (additive plus nonadditive) genetic value. We confirmed previous findings based on diallel crosses that nonadditive genetic variation is significant for key cassava traits. Specifically, we found that dominance is particularly important for root yield and epistasis contributes strongly to variation in cassava mosaic disease (CMD) resistance. Further, we showed that total genetic value predicted observed phenotypes more accurately than additive only models for root yield but not for dry matter content, which is mostly additive or for CMD resistance, which has high narrow-sense heritability. We address the implication of these results for cassava breeding and put our work in the context of previous results in cassava, and other plant and animal species.

Understanding genetic architecture requires the decomposition of genetic variance into additive, dominance, and epistatic components (Fisher 1918; Cockerham 1954; Kempthorne 1954). However, partitioning genetic variance components is notoriously difficult, requiring specialized breeding designs (*e.g.*, diallel crosses) and pedigree information (Lynch and Walsh 1998), often limiting the genetic diversity that can be sampled in any one given study. Genome-wide molecular marker data now enable the accurate measurement of relatedness in the form of genomic realized relationship matrices (GRMs; VanRaden 2008; Heffner *et al.* 2009; Lorenz *et al.* 2011). GRMs, in contrast to pedigrees, directly measure Mendelian sampling (variation in relatedness within relatedness classes such as full-siblings; Heffner *et al.* 2009). Further, GRMs can measure relationships even in diverse, nominally unrelated samples, expanding the potential for studying inheritance in natural and breeding populations (Lorenz *et al.* 2011).

Estimation of narrow-sense heritability and prediction of breeding values in genomic selection programs is becoming increasingly common using additive formulations of GRMs (Visscher *et al.* 2008). Several recent studies have described dominance and epistatic GRMs for the partitioning of nonadditive genetic variance using genome-wide SNP markers (Su *et al.* 2012; Vitezica *et al.* 2013; Muñoz *et al.* 2014; Wang *et al.* 2014). Models using these new formulations have been shown to provide improved partitioning of genetic variances relative to pedigree-based approaches (Su *et al.* 2012; Muñoz *et al.* 2014). These new models can be used not only to estimate genetic variances but also for genomic prediction of total genetic value in genomic selection breeding programs (Su *et al.* 2012; Vitezica *et al.* 2013; Muñoz *et al.* 2014; Wang *et al.* 2014).

Cassava is a vegetatively propagated, staple food crop that is high in starch and feeds half a billion people worldwide (http://faostat.fao.org). Efforts to improve cassava genetically with cutting edge methodologies including transgenic and genomic selection (GS) approaches are underway thanks to new genomic resources (Prochnik *et al.* 2012; ICGMC 2015). Prediction with additive models has recently been evaluated (Oliveira *et al.* 2012; Ly *et al.* 2013) and genomic selection using standard models is currently being tested (http://www.nextgencassava.org). Vegetatively propagated crop (*e.g.*, cassava) breeding can exploit nonadditive genetic effects by identifying superior clones as varieties (Ceballos *et al.* 2015).

Diallelic studies in cassava indicate that nonadditive genetic effects (*e.g.*, specific combining ability) are strong, particularly for root yield traits (Cach *et al.* 2005, 2006; Calle *et al.* 2005; Jaramillo *et al.* 2005; Pérez *et al.* 2005a,b; Zacarias and Labuschagne 2010; Kulembeka *et al.* 2012; Tumuhimbise *et al.* 2014; Ceballos *et al.* 2015; Chalwe *et al.* 2015). If the limited number of parents tested thus far represents the broader cassava breeding germplasm, genetic gains, especially for already low-heritability root yield traits, will be slow regardless of the breeding scheme employed (*e.g.*, phenotypic *vs.* pedigree *vs.* genomic selection). Breeding gains have indeed been slow in cassava (Ceballos *et al.* 2012) and low accuracies have been reported for genomic prediction of yield compared with cassava mosaic disease (CMD) resistance and dry matter (DM) content (Oliveira *et al.* 2012; Ly *et al.* 2013). However, cassava varieties are evaluated and disseminated to farmers by clonal propagation, meaning that accurate prediction of total (additive plus nonadditive) genetic value could contribute to variety selection.

In this study, we test whether certain cassava traits, especially root yield, have relatively large nonadditive genetic variances that account for low genomic prediction accuracies previously observed. We estimate additive and nonadditive variance components using GRMs in two datasets of cassava from the International Institute of Tropical Agriculture’s (IITA) genomic selection breeding program. Further, we assess the accuracy of predicting total genetic value using the additive and nonadditive models. We discuss the origin of nonadditive genetic variance in cassava, its potential effect on cassava breeding, and its role in genomic selection strategies for cassava improvement in the future.

## Methods

### Germplasm and phenotyping trials

We examined additive and nonadditive effects in two datasets of cassava that have been genotyped and phenotyped as part of the Next Generation Cassava Breeding Program at IITA, Nigeria (http://www.nextgencassava.org). The IITA’s Genetic Gain (GG) collection contains 694 historically important clones, most of which are advanced breeding lines although some are classified as superior landraces. These lines have been selected and maintained clonally since 1970 (Okechukwu and Dixon 2008; Ly *et al.* 2013). Most of these materials are derived from the cassava gene pool from West Africa as well as parents derived from the breeding program at Amani Station in Tanzania and hybrids of germplasm introduced from Latin America. Available information on the GG accessions included in our analyses is provided in Supplemental Material, Table S1.

IITA’s GG trials were conducted in seven locations over 14 yr (2000–2014) in Nigeria for a total of 24,373 observations. Each GG trial comprises a randomized, incomplete block design replicated one or two times per location and year. Since materials have been occasionally lost and new, selected materials are continuously added to the GG, the number of clones trialed in a given year changes gradually across years, generally increasing. The sample sizes, number of replicates, and number of clones from the GG in each of the trials (location-year combinations) are provided in Table S2.

Theory suggests that founding events and truncation selection can both lead to a conversion of nonadditive genetic variation into additive variance. This can happen because of the induction of linkage disequilibrium and reduction in allele frequency (or fixation of alleles) at some loci relative to others (Goodnight 1988; Turelli and Barton 2006; Hallander and Waldmann 2007). Consequently, our results might depend on the dataset examined. We therefore analyzed an additional dataset: a collection of 2187 clones that are the direct descendants of truncation selection on the GG. Briefly, in 2012 the GG and all available historical phenotype data were used as a reference dataset to obtain genomic estimated breeding values (GEBVs) using the genomic BLUP (GBLUP) model (VanRaden 2008; Heffner *et al.* 2009). Selection was based on an index that included mean cassava mosaic disease severity (MCMDS), mean cassava bacterial blight disease severity (MCBBS), DM, harvest index, and fresh root weight (RTWT). This index of GEBVs was used to select 83 members of the GG to cross and generate a collection of 135 full-sib families, which we refer to as the GS Cycle 1 (C1). In the C1, family sizes are 18.3 on average (median 14, range from 3 to 82). Parents have an average of 59.5 progeny (median 38, range from 5 to 406). The pedigree of the C1 is available in Table S3. Further, information about the germplasm analyzed, including data regarding the genetic structure of the population, have been published previously (Wolfe *et al.* 2016); however, we also provide plots of the first four principal components of the additive genetic relationship matrix (see below) in Figure S1.

Cycle 1 progenies were evaluated in a single clonal evaluation trial during the 2013–2014 field season across three locations (Ibadan, Ikenne, and Mokwa). For the C1 clonal trial, planting material was only available for one plot of five stands per clone, so each clone was only planted in one of the three locations (Table S2). Clones were assigned to each location so as to equally represent each family in every environment.

For both datasets, we analyzed three traits: MCMDS, DM, and RTWT. MCMDS was scored on a scale of 1 (no symptoms) to 5 (severe symptoms). We note that the distribution of MCMDS is skewed toward low disease severity (Figure S2). Most GG trials measured DM by the oven drying method although some trials used the specific gravity method. Dry matter content is expressed as a percentage of the fresh weight of roots. Fresh root weight (RTWT) is measured in kilograms per plot and is natural-log transformed to achieve normally distributed, homoscedastic residuals in all presented analyses. Trait distributions are presented in Figure S2.

### Genotype data

We used genotyping-by-sequencing (GBS) to obtain genome-wide SNP marker data (Elshire *et al.* 2011). We used the ApeKI restriction enzyme as recommended by Hamblin and Rabbi (2014). SNPs were called using the TASSEL V4 GBS pipeline (Glaubitz *et al.* 2014) and aligned to the cassava reference genome, version 5, which is available on Phytozome (http://phytozome.jgi.doe.gov) and described by the International Cassava Genetic Map Consortium (ICGMC 2015). We removed individuals with >80% missing and markers with >60% missing genotype calls. Also, markers with extreme deviation from Hardy–Weinberg equilibrium (χ^2^ > 20) were removed. If there were not at least two reads at a given locus for a given clone, the genotype was set to missing and imputed. SNP marker data were converted to the dosage format (−1 for reference-allele homozygotes, 0 for heterozygotes, and +1 for alternative-allele homozygotes) and missing data were imputed with the glmnet algorithm in R (http://cran.r-project.org/web/packages/glmnet/index.html). Similar to the approach of Wong *et al.* (2014), for each marker to be imputed, we preselected the 60 markers on the same chromosome in highest LD. We then used these preselected markers to predict missing values using the LASSO (default, *q* = 1 in glmnet), with the tuning parameter λ selected by fivefold cross-validation. We used 114,922 markers that passed these filters with a minor allele frequency >1% to construct GRMs as described below.

### Genomic relationship matrices

In order to capture additive effects variance, we constructed the genomic relationship matrix (**G**) using the formula of VanRaden (2008), method one: $G=\frac{ZZ\prime }{2\sum_{i}p_{i}q_{i}}.$ Here **Z** is a mean-centered matrix of dimension *n* individuals by *m* SNP markers. To obtain **Z**, we subtract 2(*p_i_* − 0.5) from the marker dosages, where the dosages are coded −1 for aa, 0 for Aa, +1 for AA, *p_i_* is the frequency of the second allele (A) at the *i*th locus, and *q_i_* = 1−*p_i_*. The *a* (or 0) allele refers to the reference genome allele. The **G** matrix was calculated using the *A.mat* function in the *rrBLUP* package (Endelman 2011).

We constructed a matrix to capture dominance relationships using the formulation originally proposed by Su *et al.* (2012). The dominance relationship matrix, which we will call D* (see below), is $D*=\frac{HH\prime }{\sum_{i}2p_{i}q_{i}\left(1-2p_{i}q_{i}\right)}$ where **H** is a mean-centered dominance deviation matrix with the same dimensions as **Z**. To obtain **H**, we score heterozygotes as 1 and homozygotes as 0, and subtract the mean (2*p_i_q_i_*) from the scores. We made a custom modification (available at ftp://ftp.cassavabase.org/manuscripts/) to the *A.mat* function to produce the D* matrix.

The D* dominance matrix was shown by Vitezica *et al.* (2013) to produce a partition of genetic variance appropriate for studying genetic architecture because it isolates additive effect variance from dominance effects. However, this partition is not correct for breeding purposes because the additive variance produced is not equivalent to the variation in breeding value. Vitezica *et al.* (2013) subsequently derived the matrix **D**, defined as $D=\frac{WW\prime }{\sum_{i}{\left(2p_{i}q_{i}\right)}^{2}},$ where **W** is a marker matrix with markers coded 0 for aa, 2*p_i_* for Aa, and 4*p_i_* − 2 for AA and then centered on the mean, 2*p_i_*^2^. Although our focus in the present study is not on the prediction of breeding value, the matrix **D** has been shown by Zhu *et al.* (2015) to have the advantage of being uncorrelated (under Hardy–Weinberg equilibrium) with the matrix **G**. For this reason, we tested the **D** and D* matrices and provide comparison of their results. Except where explicitly comparing matrices, we use **D** to indicate the dominance matrix D* as in Su *et al.* (2012).

Finally, we constructed relationship matrices that capture epistasis by taking the Hadamard product (element-by-element multiplication; denoted #) of matrices (Henderson 1985). For simplicity, we only explored additive-by-additive (**A#A**) and additive-by-dominance (**A#D**) relationships in this study.

### Variance component and heritability models

#### Single-step, multi-environment:

We used several approaches to estimate the relative importance of additive and nonadditive effects in the Genetic Gain and Cycle 1 datasets. In the first analysis, we analyzed the multi-year, multi-location GG data with a single-step mixed-effects model. Since the entire historical phenotype dataset is large (24,373 observations) and was relatively unbalanced in sample size across years and locations, we only analyzed data from trials with >400 individuals. This filter resulted in a dataset of 7745 observations from three locations (Ibadan, Ubiaja, Mokwa) and 8 yr (2006–2014, except 2012). All 694 genotyped GG clones were represented in this dataset (Table S2).

The models we fit were similar to those described in Ly *et al.* (2013). The full model was specified as follows: **$y=X\beta +Z_{loc.year}l+Z_{rep}r+Z_{add}a+Z_{dom}d+Z_{epi}i+\varepsilon .$** Here, ***y*** represents raw phenotypic observations. In our data, the only fixed effect (β) was an intercept for all traits except RTWT, which contained a covariate accounting for variation in the number of plants harvested per plot. The random effects terms for experimental design terms included a unique intercept for each trial (*i.e.*, location-year combination), $l\sim N\left(0,I\sigma_{l}^{2}\right),$ where **I** is the identity matrix and $\sigma_{l}^{2}$ is the associated variance component as well as a replication effect, nested in location-year combination, $r\sim N\left(0,I\sigma_{r}^{2}\right).$

The genetic variance component terms included $a\sim N\left(0,G\sigma_{a}^{2}\right),$ where **G** is the additive genetic relationship matrix and $\sigma_{a}^{2}$ is the additive genetic variance component. Similarly, $d\sim N\left(0,D\sigma_{d}^{2}\right),$ is the dominance effect with covariance **D** equal to the dominance relationship matrix and $\sigma_{d}^{2}$ equal to the dominance variance. The epistatic term $i\sim N\left(0,E\sigma_{i}^{2}\right)$ where the covariance matrix **E** took the form either of the **A#A** matrix (additive-by-additive) or the **A#D** matrix (additive-by-dominance) and the epistatic variance $\sigma_{i}^{2}$ was correspondingly either $\sigma_{A\#A}^{2}$ or $\sigma_{A\#D}^{2}.$ The final term **ε** is the residual variance, assumed to be random and distributed $N\left(0,I\sigma_{\varepsilon }^{2}\right).$ The terms **X**, **Z_loc.year_**, **Z_rep_**, **Z_add_**, **Z_dom_**, and **Z_epi_** are incidence matrices relating observations to the levels of each factor. We list the different models fit in Table 1, each of which are variations on the full model described above.

**Table 1:** Additive plus nonadditive genetic models tested and their abbreviations

Model	Relationship Matrices/Variance Components
Add	Additive
Dom	Dominance
A+D	Additive + Dominance
A×A	Additive + Dominance + A×A Epistasis
A×D	Additive + Dominance + A×D Epistasis

The formulation described above was used to fit the subset of the GG historical data described above in a single model. For the C1 progenies only a single season was available and therefore we fit all data together in a single model. Since the C1 trials were conducted across three locations but with no replications we fit the same model for C1 as GG excluding the replication term. The models described above were fit using the *regress* package in R (Clifford and McCullagh 2006). The *regress* function finds restricted maximum likelihood (REML) solutions to mixed models using the Newton–Raphson algorithm.

For each trait, in both the C1 and GG we identified a “best fit” model among the models listed in Table 1, based on the lowest Akaike Information Criterion [AIC; 2*k – 2*ln(*likelihood*), where *k* = number of parameters estimated]. In addition, we calculated the Bayesian Information Criterion [BIC; −2*ln(likelihood) + k*ln(*n*), where *n* = number of observations and *k* = number of parameters estimated]. We also examined the log-likelihood of each model and the proportion of variance explained by genetic factors (*H*^2^). The precision of variance component estimates and the dependency among estimates was examined using the asymptotic variance–covariance matrix of estimated parameters, provided by *regress* (**V**). We report standard errors for each variance component, defined as the square root of the diagonal of **V**. We also converted **V** into a correlation matrix [**F**, as in Muñoz *et al.* (2014)], where **F** is defined as **L^−1/2^VL^−1/2^** and **L** is a diagonal matrix containing one over the square root of the diagonal of **V**. We use **F** to assess the dependency of variance components estimates, especially for comparing results among traits and datasets.

#### Within-trial analyses:

We used only a subset of the GG trials to estimate variance components in the single-step multi-environment model described above. In addition, we were able to analyze the entire historical GG data by testing each trial (*N* = 47, unique location-year combinations) separately. This provided us with 47 estimates of additive, dominance, and epistatic variance. We examine the distribution of variance components estimates. As in the multi-environment models, within-trial models were fit with *regress* in R.

### Genomic prediction and cross-validation

We assessed the influence that modeling nonadditive genetic variance components have on genomic prediction using a cross-validation strategy. Because single-step multi-environment models are computationally intensive, we used a two-step approach here. In the first step, we combined data from all available GG and C1 trials using the following mixed model: $y=X\beta +Z_{rep}r+Z_{clone}g+\varepsilon .$ In this model, **β** included a fixed effect for the population mean, the location-year combination, and for RTWT only, the number of plants harvested per plot. As in the single-step, multi-environment model for GG, we included the random replication effect $r\sim N\left(0,I\sigma_{r}^{2}\right).$ In contrast to the previous model, we did not at this stage include a genomic relationship matrix, instead we fit a random effect for clone, $g\sim N\left(0,I\sigma_{g}^{2}\right),$ where the covariance structure was the identity matrix, **I**. The BLUP ($\hat{g}$) for the clone effect therefore represents an estimate of the total genetic value for each individual. The mixed model above was solved using the *lmer* function of the *lme4* R package (Bates *et al.* 2014).

In our data, the number of observations per clone ranges from 1 to 131 with median of 2 and mean of 5.97 excluding the checks TMEB1 and I30572, which had 941 and 902 observations, respectively. Pooling information from multiple years and locations, especially when there is so much variation in numbers of observations can introduce bias. Much theory, particularly in animal breeding has been developed to address this issue, and we followed the approach recommended by Garrick *et al.* (2009). Briefly, BLUPs ($\hat{g}$) for clone were deregressed according to $\hat{g}/r^{2}$ where ***r*^2^** is the reliability $\left(1-\;\frac{PEV}{\sigma_{g}^{2}}\right)$ and PEV is the prediction error variances of the BLUP. In the second step of analysis, where deregressed BLUPs are used as response variables, weights are applied to the diagonal of the error variance–covariance matrix **R**. Weights are calculated as $\frac{1-h^{2}}{0.1+\frac{1-{\text{r}}^{2}}{r^{2}}h^{2}},$ where *h^2^* is the proportion of the total variance explained by the clonal variance component, $\sigma_{g}^{2}$ (Garrick *et al.* 2009).

We implemented a fivefold cross-validation scheme replicated 25 times to test the accuracy of genomic prediction using the GRMs and models described above (Table 1). In this scheme, for each replication, we randomly divided the dataset into five equally sized parts (*i.e.*, folds). We used each fold in turn for validation by removing its phenotypes from the training population and then predicting them. We calculated accuracy as the Pearson correlation between the genomic prediction and the BLUP ($\hat{g}$, not-deregressed) from the first step. For each model, we calculated accuracy of the prediction for total genetic value, defined as the sum of the predictions from all available kernels (*e.g.*, additive + dominance + epistasis). Genomic predictions were made using the *EMMREML* R package (Akdemir and Okeke 2015).

### Data availability

All raw genotype and phenotype data are available at ftp://ftp.cassavabase.org/manuscripts/ along with custom code used to make deregressed BLUPs, conduct fold cross-validation, and calculate dominance relationship matrices.

## Results

### Partitioning the genetic variance: single-step, multi-environment models

We used several approaches to estimate genetic variance components in our dataset. The first was to fit single-step models to two datasets: the Genetic Gain (GG) and the Cycle 1 (C1). For each trait, in each dataset, we first identified the best fitting model of the five tested (Table 1) on the basis of lowest AIC. Model comparisons based on AIC and BIC are summarized in Table 2. Key results from the best models for both GG and C1 are summarized in Table 3 with more detailed results from all models provided in Table S4 and Table S5.

**Table 2:** Comparison of models by AIC and BIC

		Genetic Gain (GG)		Cycle 1 (C1)		Genetic Gain (Within Trials)	
Trait	Model	AIC	BIC	AIC	BIC	AIC	BIC
DM	Add	**18,921.9**	**18,947.9**	7094.3	7110.8	**1335.9 ± 140.3**	**1355.2 ± 141.0**
	Dom	18,947.7	18,973.7	7176.6	7193.2	1338.6 ± 140.7	1357.8 ± 141.4
	A+D	18,922.4	18,954.9	**7083.9**	**7106.0**	1337.4 ± 140.3	1360.2 ± 141.1
	A×A	18,923.0	18,962.0	7085.0	7112.6	1339.0 ± 140.3	1365.2 ± 141.2
	A×D	18,922.6	18,961.6	7085.9	7113.5	1339.0 ± 140.2	1365.3 ± 141.1
RTWT	Add	−4716.1	−4688.5	−315.2	−298.0	−310.7 ± 42.9	−287.1 ± 42.6
	Dom	−4731.0	−4703.4	−**361.0**	−**343.8**	−311.9 ± 42.5	−**288.3 ± 42.2**
	A+D	−4740.8	−**4706.2**	−360.4	−337.4	−**311.9 ± 42.9**	−284.3 ± 42.6
	A×A	−**4744.2**	−4702.7	−358.4	−329.7	−311.0 ± 43.0	−279.4 ± 42.6
	A×D	−4743.6	−4702.1	−358.4	−329.7	−311.1 ± 43.0	−279.5 ± 42.6
MCMDS	Add	1255.7	1283.4	2417.8	2435.3	38.2 ± 47.1	62.1 ± 47.2
	Dom	1207.6	1235.4	2746.4	2763.8	20.1 ± 47.3	**44.1 ± 47.4**
	A+D	1202.1	1236.9	2396.3	2419.5	20.8 ± 47.3	48.8 ± 47.4
	A×A	1180.7	1222.4	2391.7	2420.8	19.6 ± 47.0	51.7 ± 47.1
	A×D	**1173.0**	**1214.7**	**2378.9**	**2408.0**	**18.1 ± 47.0**	50.2 ± 47.2

For each trait, five genetic models are compared based on Akaike’s Information Criterion (AIC) and the Bayesian Information Criterion (BIC). Comparisons are done based on single-step multi-environmental models for the Genetic Gain (GG) and Cycle 1 (C1) datasets. In addition, the mean and standard error AIC/BIC from 47 GG trials, each analyzed separately, are provided. For each dataset and each trait the lowest AIC and BIC are bolded.

**Table 3:** Best fitting single-step multi-environment model results

Dataset	Genetic Gain (GG)			Cycle 1 (C1)		
Trait	DM	RTWT	MCMDS	DM	RTWT	MCMDS
Best model	Add	A×A	A×D	A+D	Dom	A×D
σ^2^_loc.year_	0.025	0.056	0.051	8.38	0.006	0.054
	(4.8)	(0.03)	(0.02)	(8.4)	(0.007)	(0.055)
σ^2^_rep_	6.16	0.014	0.000	—	—	—
	(5.4)	(0.01)	(0)	—	—	—
σ^2^_add_	10.44	0.029	0.32	17.3	—	1.780
	(1)	(0.012)	(0.1)	(2.5)	—	(0.178)
σ^2^_dom_	—	0.020	0.000	3.4	0.116	0.172
	—	(0.011)	(0.08)	(1.5)	(0.018)	(0.082)
σ^2^_epi_	—	0.033	0.556	—	—	0.514
	—	(0.014)	(0.09)	—	—	(0.101)
σ^2^_error_	15.36	0.17	0.34	10.7	0.25	0.26
	(0.33)	(0.003)	(0.006)	(0.7)	(0.011)	(0.023)
*h*^2^	0.33	0.09	0.25	0.43	—	0.64
*d*^2^	—	0.06	0.00	0.08	0.31	0.06
*i*^2^	—	0.10	0.44	—	—	0.18
*H*^2^	0.33	0.25	0.69	0.52	0.31	0.89
loglik	−9457	2378.1	−581	−3538	184	−1184

Variance components (± SEs), narrow-sense heritabilities (*h*^2^), proportion of the total phenotypic variance explained by dominance (*d*^2^), epistasis (*i*^2^_epi_), and broad-sense heritability (*H*^2^) are provided. Model log-likelihoods are also given. The models shown were selected on the basis of having the lowest Akaike Information Criterion (AIC) relative to other tested models.

The AIC-selected best model for MCMDS included additive-by-dominance epistasis (A×D) in both GG and C1. For RTWT, the model with additive-by-additive epistasis (A×A) fit best in the GG but a simpler dominance only (Dom) model was selected in the C1 dataset. Finally, for DM the additive only (Add) model was best in the GG but additive plus dominance (A+D) was selected in the C1 dataset. The BIC criterion places a steeper penalty on increasing the number of parameters. Nevertheless, BIC selected the same model as AIC in all cases except for RTWT in the GG dataset, where the additive plus dominance model was preferred (Table 2, Table S4, and Table S5). Based on the guidelines of Raftery (1995), the evidence that the best models for RTWT include dominance and models for MCMDS include nonadditive effects, especially epistasis, is very strong (>10 AIC/BIC difference).

**Figure 1:**
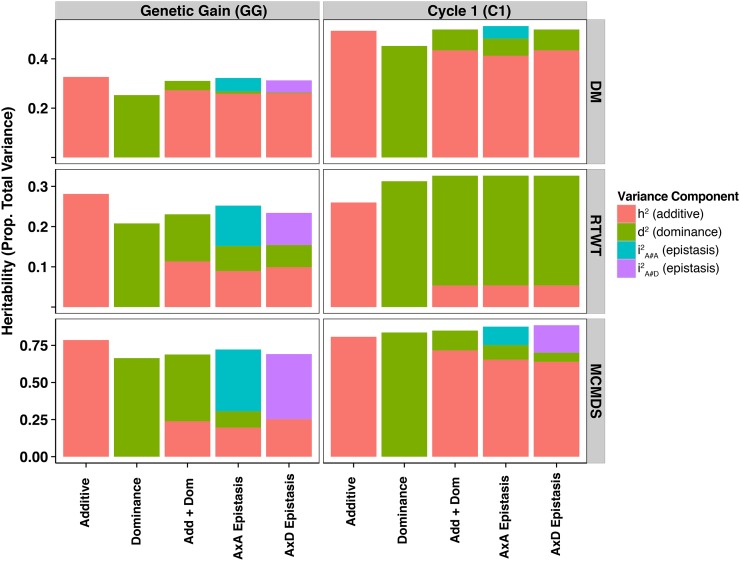
Partitioning of broad-sense heritability for single-step multi-environment models in the Genetic Gain and Cycle 1 datasets. Results from each of five models are shown in each panel broken down by trait (rows) and population (columns). Models include additive only (Additive), dominance only (Dominance), Additive plus Dominance (Add + Dom), Additive plus dominance plus either A×A epistasis (A×A Epistasis) or A×D epistasis (A×D Epistasis).

We noted that for every trait, when comparing the model achieving the highest broad-sense heritability (*H*^2^), the *H*^2^ was higher in C1 compared to GG. This can be seen most easily in Figure 1, which shows how total explainable genetic variance (*H*^2^) is partitioned among variance components in the C1 and GG (also see Tables 3, Table S4, and Table S5). We also noted that the additive only model had the highest *H*^2^ for all traits in the GG dataset, but C1 models with nonadditive components always had at least slightly higher *H*^2^.

On the basis of genetic variance captured, DM had *H*^2^ between 0.25 and 0.53 and had mostly additive inheritance across all models (Figure 1, Table 3, Table S4, and Table S5). In contrast, nonadditive components accounted for the majority of genetic variance for RTWT, with *H*^2^ between 0.21 and 0.33. Dominance was significant in all models tested for RTWT in both datasets and epistasis was significant in the GG dataset. MCMDS had the highest *H*^2^ compared to the other traits (0.66–0.89) and was similar to RTWT in that dominance and/or epistasis were always significant where included. While nonadditive genetics were the majority of *H*^2^ in GG, they were much less important in C1 for MCMDS.

We examined the asymptotic correlation matrices of parameter estimates (**F**) to ascertain the dependency of variance component estimation. The correlation between genetic variance components was always negative and was, in general, of greater magnitude in the GG compared to the C1 (Table S6, Table S7, Table S8, Table S9, Table S10, and Table S11). Correlations between additive and dominance components were greatest in the A+D models (range −0.81 to −0.83 in the GG and −0.5 to −0.61 in the C1). Correlations between additive and dominance components dropped in models with epistasis (range −0.42 to −0.63, GG and −0.26 to −0.58, C1). Correlations between additive and A×A epistatic variances (range −0.09 to −0.29) and A×D epistasis (range −0.07 to −0.22) were low. Correlations between dominance components and epistasis were higher, ranging from −0.28 to −0.64 with A×A epistasis and −0.36 to −0.69 with A×D epistasis.

Comparison of the two alternative dominance matrices D* (results described above) and **D** revealed very similar results. In almost every case, AIC and BIC selected the same best fit model for **D** and D*. The exception was RTWT in the C1 dataset where the A+D model was preferred over the dominance only model when using the **D** matrix instead of D* (Table S4 and Table S5). The AIC and BIC are on the whole slightly lower for the models using the **D** matrix, indicating a better fit to the data. As expected, the correlation between additive and dominance parameter estimates is of smaller magnitude for all analyses with **D** compared with D* (Table S6, Table S7, Table S8, Table S9, Table S10, and Table S11). However, the correlation between additive and epistatic as well as between dominance and epistatic variances is always of greater magnitude with **D**. Finally and as expected, models using the **D** matrix generally explain the same amount of genetic variance as those with D* but partition a smaller portion to dominance (Figure S3). We noted that for RTWT in the C1 dataset, models with **D** actually achieve a slightly higher broad-sense heritability. Because of the similarity of results, we focused the remainder of our analyses and discussion on the results from the D* matrix, henceforth referred to only as **D**.

### Partitioning the genetic variance: within-trial analyses

**Figure 2:**
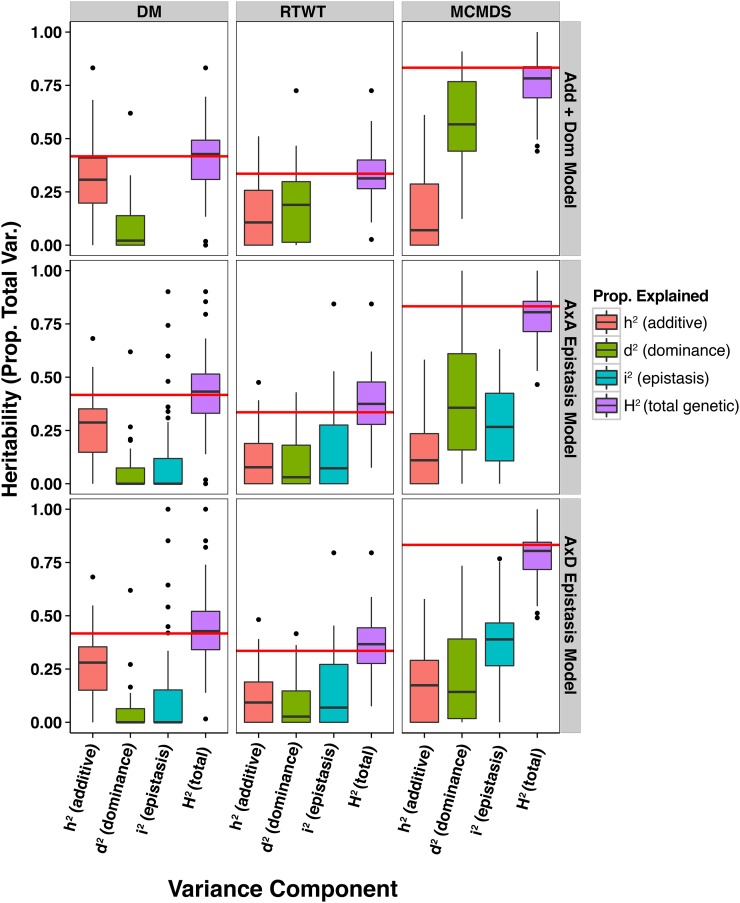
Distribution of genetic variance proportions across GG trials. Three models were fitted for each trait in each of 47 GG trials. Each panel contains boxplots showing the distribution of proportions of the phenotypic variability explained by a corresponding genetic factor, including the broad-sense heritability (*H*^2^). Red horizontal lines are the median narrow-sense heritability (*h*^2^) from the additive only model. Traits are on columns and three models are on the rows: additive plus dominance (Add + Dom), additive plus dominance plus A×A epistasis (A×A Epistasis), and additive plus dominance plus A×D epistasis (A×D Epistasis).

We also examined variance partitioning within each of 47 GG trials for the five models described in Table 1. This provided a means of testing the entire available dataset for nonadditive variances, in contrast to the multi-environment models described above. The mean and variability of model parameters (variance components, heritability, *etc*.) across these trials are summarized in Table S12 and results for each individual trial-model combination are given in Table S13. Figure 2 provides a visual summary of the proportion of phenotypic variability explained by each genetic variance component on average across the trials. We also compared the mean AIC across trials (Table 2 and Table S10) and found them to agree overall with the results of the one-step multi-environment models (Table 3). Specifically, the models that fit best in the one-step models were best on average in the within-trial analyses for DM (Add) and MCMDS (A×D). However, for RTWT the within-trial AIC-best model was A+D compared to A×D in the one-step multi-environment model. In contrast to the one-step multi-environment model, the BIC agreed with AIC only for DM. For RTWT and MCMDS the simpler dominance only model was preferred by BIC on average (Table 2).

### Genomic prediction of additive and total genetic value

**Figure 3:**
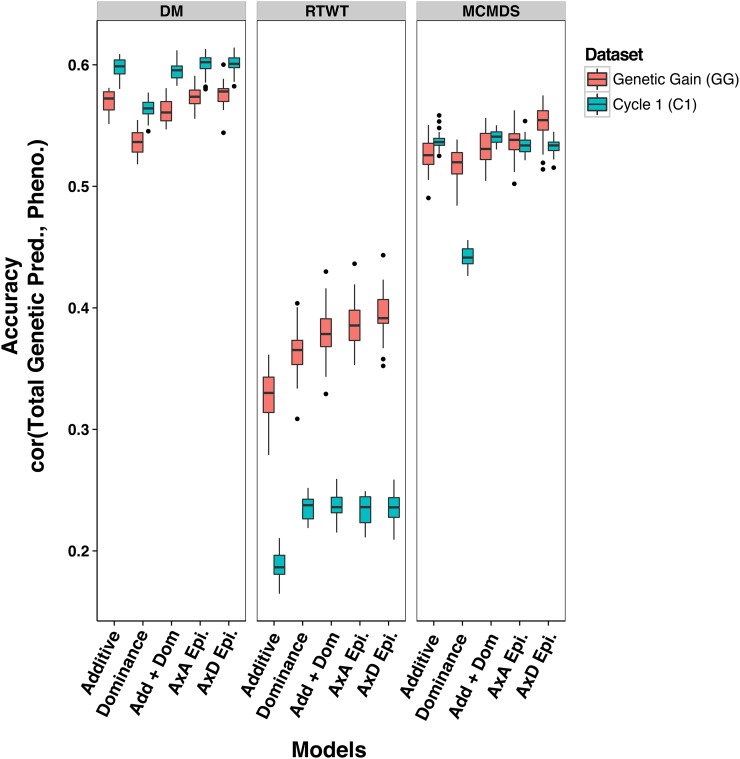
Accuracy of total genetic value prediction in the Genetic Gain and Cycle 1 datasets. Boxplots showing the distribution over 25 replicates of fivefold cross-validation of the prediction accuracy of the total genetic value from five different models are shown in each panel. The accuracy within the Genetic Gain (red) and Cycle 1 (blue) are shown. Traits are in the columns. Accuracy is defined as the correlation between the sum of predictions from all genetic variance components in the model and the BLUP from the first stage of analysis where location, year, and replicate variability were removed. Models included are: additive only (Additive), dominance only (Dominance), additive plus dominance (Add + Dom), additive plus dominance plus A×A epistasis (A×A Epi.), and additive plus dominance plus A×D epistasis (A×D Epi.).

We used cross-validation to assess the prediction accuracy for total genetic value from the five models (Table 1) in both datasets. Compared to the single-kernel additive prediction using the additive relationship matrix, multi-kernel total genetic value predictions were an average of 7% better (maximum of 26% improvement; Figure 3, Table S14, and Table S15). By model, improvements in the correlation between total value and phenotype over the additive only model were 7, 7, and 8% for A+D, A×A, and A×D, respectively. The additive only model predictions were on average 12% less accurate in the C1 than in the GG. Total genetic value predictions were less accurate by 12% in the C1 relative to GG. The models we fit for genomic prediction involved the estimation by *EMMREML* of weights, used to create a single kernel that is the weighted average of multiple original kernels and corresponding to the partitioning of genetic variance among the kernels. The average total weight given to nonadditive components for both DM and MCMDS was 0.41 but was 0.92 for RTWT.

## Discussion

In clonally propagated crops, nonadditive genetic effects can be effectively exploited by the identification of superior genetic individuals as varieties. For this reason, we quantified the amount and nature of nonadditive genetic variation for key traits in a genomic selection breeding population of cassava from sub-Saharan Africa. We then assessed the accuracy of genomic prediction of additive compared to total (additive plus nonadditive) genetic value. Using several approaches and datasets based on genome-wide marker data, we confirmed previous findings in cassava based on diallel crosses nonadditive genetic variation is significant, especially for yield traits (Cach *et al.* 2005, 2006; Calle *et al.* 2005; Jaramillo *et al.* 2005; Pérez *et al.* 2005a,b; Zacarias and Labuschagne 2010; Kulembeka *et al.* 2012; Tumuhimbise *et al.* 2014; Ceballos *et al.* 2015; Chalwe *et al.* 2015). A potential weakness of the marker system we used (GBS) is that it generates a high proportion of missing marker data and it may undercall heterozygotes when read depth is insufficient. The similarity of our findings to previous research, and the important difference in the observations on RTWT *vs.* DM, however, suggest that this weakness did not strongly affect our results. Further, we found that multi-component models incorporating nonadditive effects predict observed phenotypes more accurately than additive only models for root yield but not for DM content, which has primarily additive inheritance, or for CMD resistance, which has high narrow-sense heritability. We address the implication of these results for cassava breeding and put our work in the context of previous results in cassava, other plant, and animal species below.

Our results indicate strong nonadditive (mainly dominance) variance for root yields and mostly additive inheritance of root DM content. These findings confirm the conclusions of numerous diallelic studies conducted with both Latin American (Cach *et al.* 2005, 2006; Calle *et al.* 2005; Jaramillo *et al.* 2005; Pérez *et al.* 2005a,b) and African cassava (Zacarias and Labuschagne 2010; Kulembeka *et al.* 2012; Tumuhimbise *et al.* 2014; Chalwe *et al.* 2015) germplasm (see also Ceballos *et al.* 2015). In agreement with the findings of Ly *et al.* (2013), we found cassava mosaic disease severity (MCMDS) to be well predicted with an additive only model. However, we found significant dominance and epistatic components in both populations analyzed. This result is in line with previous diallelic studies indicating significant SCA (Tumuhimbise *et al.* 2014; Chalwe *et al.* 2015) and genetic mapping studies that identified a single major effect QTL with a dominant CMD resistance effect (Akano *et al.* 2002; Okogbenin *et al.* 2012; Rabbi *et al.* 2014). In addition, a recent genome-wide association and prediction study of MCMDS using nonadditive GRMs found that dominance and especially epistasis explain most of the variance in the region of a large-effect QTL, suggesting multiple interacting loci in the region (Wolfe *et al.* 2016).

The importance of nonadditive genetic variance in evolution by natural and artificial selection is controversial (Hill *et al.* 2008; Crow 2010; Hansen 2013). Nevertheless, numerous studies have found and exploited dominance and epistasis in animal breeding, including dairy (Ahlborn-Breier and Hohenboken 1991; Fuerst and Sölkner 1994; Varona *et al.* 1998; Van Tassell *et al.* 2000; Palucci *et al.* 2007) and beef (Rodriguezalmeida *et al.* 1995) cattle. Diallelic studies have indicated significant SCA for maize grain yield (Doerksen *et al.* 2003; Wardyn *et al.* 2007). Aside from cassava, breeding of other noninbred, clonally propagated species also identifies and makes use of nonadditive effects, including in potato (Killick 1977), *Eucalyptus* (Costa *et al.* 2004), and loblolly pine (Muñoz *et al.* 2014). More recently, marker-based and GRM-based models have identified significant nonadditive effects in pigs (Su *et al.* 2012; Nishio and Satoh 2014), mice (Vitezica *et al.* 2013), beef cattle (Bolormaa *et al.* 2015), dairy cows (Morota *et al.* 2014), maize (Dudley and Johnson 2009), soy (Hu *et al.* 2011), loblolly pine (Muñoz *et al.* 2014), and apple (Kumar *et al.* 2015). Results from the present study suggest that accounting for nonadditive effects in the variety development pipeline should increase the value of hybrids released by cassava breeding programs.

One of the more interesting aspects of our study relative to previous ones is the comparison between a parental generation (the Genetic Gain) and their offspring (Cycle 1), a collection of full- and half-sib families. From GG to C1, the *H*^2^ generally increased. For RTWT, this is largely attributable to increased nonadditive variance in contrast to MCMDS where the increase is concomitant with a drop in nonadditive variance. In contrast to our result, the theory suggests that reduction (or fixation) of allele frequencies at some loci relative to others in populations undergoing bottlenecks (Goodnight 1988), inbreeding (Turelli and Barton 2006), or truncation selection (Hallander and Waldmann 2007) should cause a conversion of nonadditive (where present) to additive variance. These results have, however, been based on models with finite numbers of loci in linkage equilibrium. Based on the mean diagonal of the additive genetic relationship matrix, C1 (0.66) does not appear notably more inbred than GG (0.64). We also calculated mean pairwise LD (GG = 0.27, C1 = 0.29) and mean LD block size (21.7 kb in GG and 23.1 kb in C1) using standard settings in PLINK (version 1.9, https://www.cog-genomics.org/plink2) and found the two generations to be similar.

Probably the strongest explanation for the difference in genetic variance components between GG and C1 is the family structure (135 full-sib families from 83 outbred parents). In a population of full-sibs three quarters of the dominance variance is expressed within families and all of it for half-sib populations (Hallauer *et al.* 2010; Ceballos *et al.* 2015). Indeed, increasing the number of full-sib relationships is known to increase the nonadditive genetic variance detectable in a population (Varona *et al.* 1998; Van Tassel *et al.* 2010).

It is also conceivable that maternal plant effects could increase apparent nonadditive effects in C1. The C1 clones in contrast to the GG clones are new, and were derived from stem cuttings of seedling plants germinated in the previous field season (2012–2013). The suggestion is therefore that the quality and vigor of the seedling plant giving rise to the C1 clones may influence their performance in the 2013–2014 trial. We further caution that comparison of GG and C1 may be biased by the disproportionate amount of data from different locations and years available for the GG.

In our study, when additive and nonadditive kernels were used together, the variance explained by the additive component, particularly for RTWT, decreased. One interpretation of this result is that the additive component alone absorbs some nonadditive variance. Similar results have been obtained by other researchers, leading to similar conclusions (Lu *et al.* 1999; Su *et al.* 2012; Zuk *et al.* 2012; Muñoz *et al.* 2014). We note that this phenomenon occurs whether we use the D* matrix, which is correlated with the **G** matrix, or the **D** matrix, which is theoretically orthogonal to **G**. We suggest therefore that prediction models that do not explicitly incorporate nonadditive components may achieve gains in the short term that break down over the long term (Cockerham and Tachida 1988; Walsh 2005; Hansen 2013). Our prediction tests in this study were focused on total genetic values and used the D* matrix. However, we hypothesize that including nonadditive GRMs, particularly the **D** matrix, when estimating additive genetic (*i.e.*, breeding) values would provide a less biased, more accurate selection of parents for crossing.

Nonadditive variation is prevalent in cassava, especially for low-heritability traits. This has many important implications for cassava breeding. It explains, in part, why GGs have been slow (Ceballos *et al.* 2012). Inbreeding to convert dominance variance to additive and better control epistatic combinations, as in maize, has been suggested as a solution to nonadditive genetics (Ceballos *et al.* 2015). Even for low *h*^2^ traits and without inbred cassava, using the kinds of models presented in this paper, good parents can be selected based on additive predictions and total genetic value can be predicted for the identification of potential commercial varieties, all based on the combination of marker and preliminary field trial data (Heslot *et al.* 2015). This approach has been previously advocated for plant breeding (Oakey *et al.* 2007; Heslot *et al.* 2015) and has proven effective in animal breeding (*e.g.*, Ahlborn-Breier and Hohenboken 1991; Palucci *et al.* 2007; Su *et al.* 2012; Nishio and Satoh 2014). Nonadditive models using GRMs can thus improve the efficiency and productivity of variety selection pipelines that are the most labor- and time-intensive part of selecting good cassava clones after crossing.

## Supplementary Material

Supplemental Material
