# Supplementary material for: Marker-Based Estimates Reveal Significant Nonadditive Effects in Clonally Propagated Cassava (Manihot esculenta): Implications for the Prediction of Total Genetic Value and the Selection of Varieties
Source: G3 (Bethesda). 2016 Aug 30;6(11):3497–506. doi: 10.1534/g3.116.033332 (PMC5100848; doi:10.1534/g3.116.033332)
Supplement: Supplemental Material [file supp_6_11_3497__index.html]

Marker-Based Estimates Reveal Significant Nonadditive Effects in Clonally Propagated Cassava (Manihot esculenta): Implications for the Prediction of Total Genetic Value and the Selection of Varieties — Supplemental Material 

# Marker-Based Estimates Reveal Significant Nonadditive Effects in Clonally Propagated Cassava (*Manihot esculenta*): Implications for the Prediction of Total Genetic Value and the Selection of Varieties

## Supplemental Material for Wolfe *et al.*, 2016

**Files in this Data Supplement:**

- Figure S1 - Genetic structure of the IITA Genetic Gain germplasm (red) and the Cycle 1 progenies (blue). (.pdf, 175 KB)
- Figure S2 - Distribution of raw (left) and BLUP (right) phenotypes. (.pdf, 71 KB)
- Figure S3 - Comparison between the partitioning of broad-sense heritability for models using two alternative dominance matrices, D and D\* in the Genetic Gain and Cycle 1 datasets. (.pdf, 129 KB)
- Table S1 - Pedigree and related information for the IITA: Genetic Gain germplasm analyzed in this study. (.xlsx, 38 KB)
- Table S2 - Details on design of field trials analyzed. (.xlsx, 11 KB)
- Table S3 - Pedigree information for the IITA: GS Cycle 1 germplasm analyzed in this study. (.xlsx, 67 KB)
- Table S4 - Results from fitting five different additive and non-additive genetic mixed-models for three key cassava traits in a single-step to data from multiple locations and years for the IITA Genetic Gain dataset. (.xlsx, 23 KB)
- Table S5 - Results from fitting five different additive and non-additive genetic mixed-models for three key cassava traits in a single-step to data from multiple locations for the IITA Cycle 1 dataset. (.xlsx, 21 KB)
- Table S6 - The asymptotic correlation matrices of parameter estimates for each trait from an additive plus dominance genetic model fit in the IITAï¿½s Genetic Gain dataset. (.xlsx, 10 KB)
- Table S7 - The asymptotic correlation matrices of parameter estimates for each trait from an additive plus dominance plus additive-by-additive epistasis genetic model fit in the IITAï¿½s Genetic Gain dataset. (.xlsx, 11 KB)
- Table S8 - The asymptotic correlation matrices of parameter estimates for each trait from an additive plus dominance plus additive-by-dominance epistasis genetic model fit in the IITAï¿½s Genetic Gain dataset. (.xlsx, 11 KB)
- Table S9 - The asymptotic correlation matrices of parameter estimates for each trait from an additive plus dominance genetic model fit in the IITAï¿½s Cycle 1 dataset. (.xlsx, 10 KB)
- Table S10 - The asymptotic correlation matrices of parameter estimates for each trait from an additive plus dominance plus additive-by-additive epistasis genetic model fit in the IITAï¿½s Cycle 1 dataset. (.xlsx, 11 KB)
- Table S11 - The asymptotic correlation matrices of parameter estimates for each trait from an additive plus dominance plus additive-by-dominance epistasis genetic model fit in the IITAï¿½s Cycle 1 dataset. (.xlsx, 11 KB)
- Table S12 - Summary of results from five additive and non-additive genetic mixed-models for three traits across 47 trials conducted on the IITA Genetic Gain germplasm. (.xlsx, 17 KB)
- Table S13 - Results from five additive and non-additive genetic mixed-models for three traits across 47 trials conducted on the IITA Genetic Gain germplasm. (.xlsx, 120 KB)
- Table S14 - Results from 25 replicates of 5-fold cross-validation in the Genetic Gain population for three traits and five additive and non-additive genetic mixed-models. (.xlsx, 18 KB)
- Table S15 - Results from 25 replicates of 5-fold cross-validation in the Cycle 1 population for three traits and five additive and non-additive genetic mixed-models. (.xlsx, 20 KB)
