# Supplementary figures and images for: Marker-Based Estimates Reveal Significant Nonadditive Effects in Clonally Propagated Cassava (Manihot esculenta): Implications for the Prediction of Total Genetic Value and the Selection of Varieties
Source: G3 (Bethesda). 2016 Aug 30;6(11):3497–506. doi: 10.1534/g3.116.033332 (PMC5100848; doi:10.1534/g3.116.033332)

**Figure S2.** Distribution of raw (left) and BLUP (right) phenotypes.

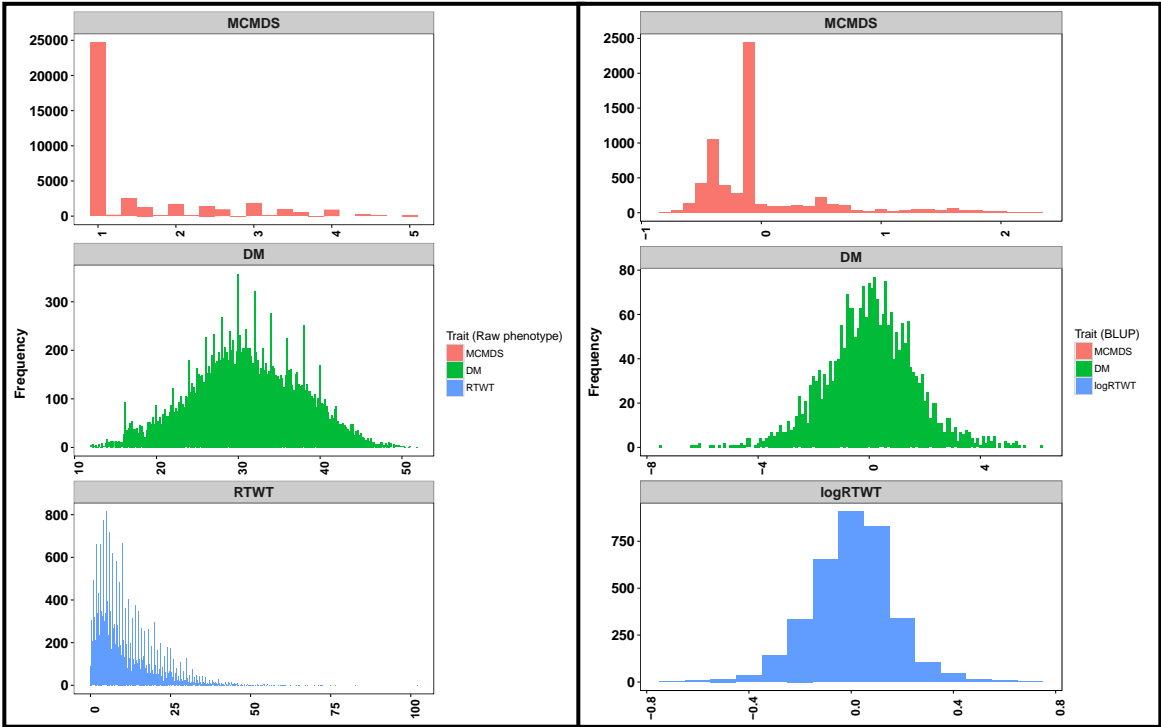

Supplement: Supplemental Material [file supp_g3.116.033332_FigureS2.pdf]
