# Supplementary material for: Marker-Based Estimates Reveal Significant Nonadditive Effects in Clonally Propagated Cassava (Manihot esculenta): Implications for the Prediction of Total Genetic Value and the Selection of Varieties
Source: G3 (Bethesda). 2016 Aug 30;6(11):3497–506. doi: 10.1534/g3.116.033332 (PMC5100848; doi:10.1534/g3.116.033332)

Figure S3. Comparison between the partitioning of broad-sense heritability for models using two alternative dominance matrices, D and D* in the Genetic Gain and Cycle 1 datasets. Results from each of five models are shown in each panel broken down by trait (rows) and population (columns). Models include additive only (Add), dominance only (Dom), Additive plus Dominance (AplusD), Additive plus dominance plus either AxA epistasis (AxA_epi) or AxD epistasis (AxD_epi). Models with dominance terms that used the **D** matrix of Vitezica et al. 2013 are distinguished from models using the **D*** matrix of Su et al. 2012 using either "*" or else "(with **D***)".

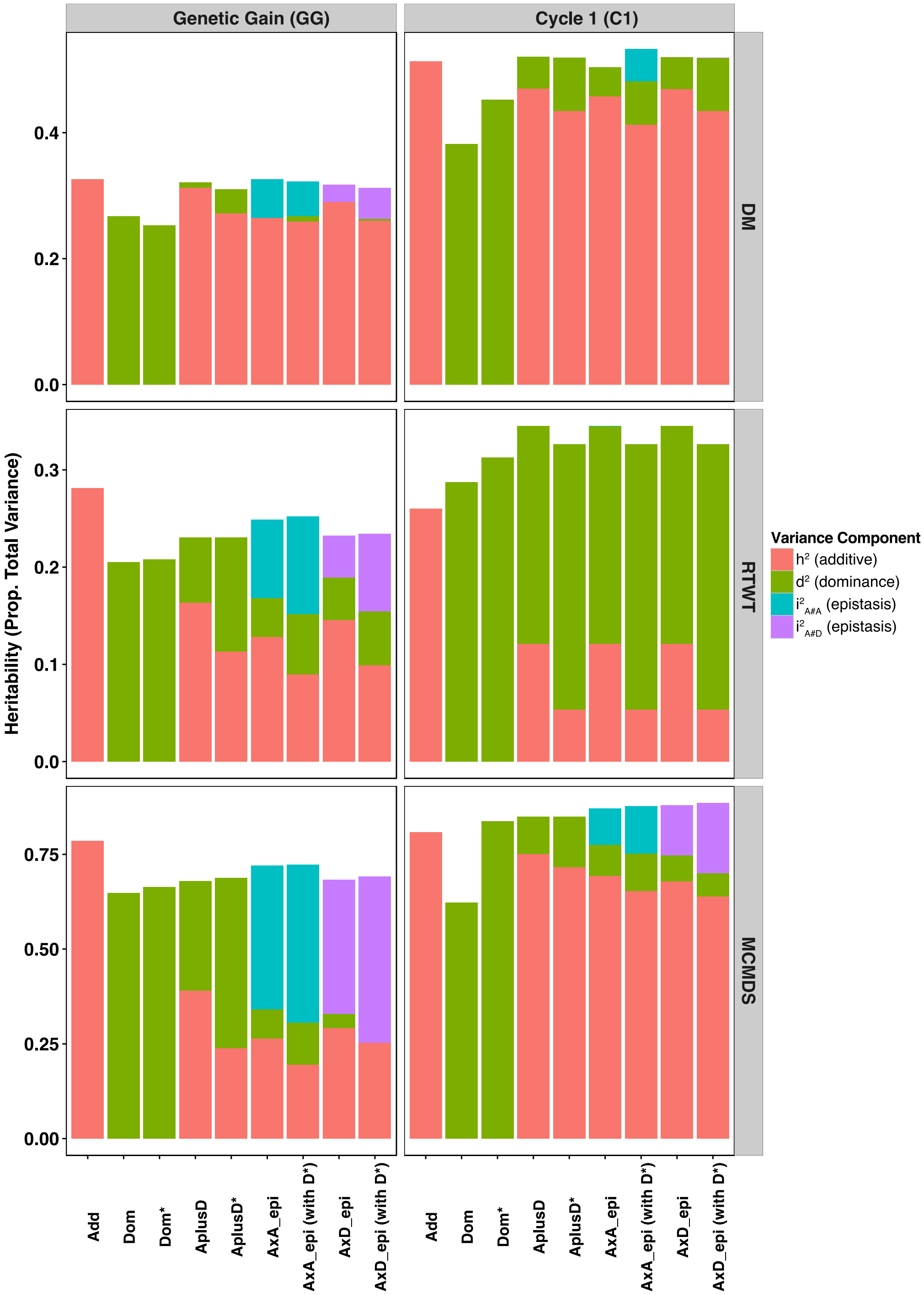

Supplement: Supplemental Material [file supp_g3.116.033332_FigureS3.pdf]
